# Expression of Serum and Exosomal microRNA-34a in Subjects with Increased Fat Mass †

**DOI:** 10.3390/ijms27010270

**Published:** 2025-12-26

**Authors:** Jacqueline Alejandra Noboa-Velástegui, Rodolfo Iván Valdez-Vega, Jorge Castro-Albarran, Perla Monserrat Madrigal-Ruiz, Ana Lilia Fletes-Rayas, Sandra Luz Ruiz-Quezada, Martha Eloisa Ramos-Márquez, José de Jesús López-Jiménez, Iñaki Álvarez, Rosa Elena Navarro-Hernández

**Affiliations:** 1 Departamento de Biología Celular, Fisiología e Inmunología, Institut de Biotecnologia i Biomedicina, Campus de Bellaterra, Bellatera, 08193 Barcelona, Spain; jacquelinealejandra.noboa@autonoma.cat; 2 Programa de Doctorado en Ciencias Biomédicas, Centro Universitario de Ciencias de la Salud, Universidad de Guadalajara, Calle Sierra Mojada No. 950, Colonia Independencia, Guadalajara C.P. 44340, Mexico; rodolfo.valdez2326@alumnos.udg.mx; 3 Departamento de Ciencias de la Salud y Ecología Humana, División de Desarrollo Regional, Centro Universitario de la Costa Sur, Autlan de Navarro C.P. 48900, Mexico; jorge.castro@academicos.udg.mx; 4 UDG-CA-701, Inmunometabolismo en Enfermedades Complejas y Envejecimiento, Departamento de Biología Molecular y Genómica, Centro Universitario de Ciencias de la Salud, Universidad de Guadalajara, Calle Sierra Mojada No. 950, Colonia Independencia, Guadalajara C.P. 44340, Mexico; perla.madrigal@academicos.udg.mx; 5 Instituto de Investigación en Enfermería y Salud Traslacional, Departamento de Enfermería Aplicada, Centro Universitario de Ciencias de la Salud, Universidad de Guadalajara, Calle Sierra Mojada No. 950, Colonia Independencia, Guadalajara C.P. 44340, Mexico; lilia.fletes@academicos.udg.mx; 6 Departamento de Farmacobiología, Division de Ciencias Básicas, Centro Universitario de Ciencias Exactas e Ingenierias, Universidad de Guadalajara, Blvd. Gral. Marcelino García Barragán No. 1421, Colonia Olímpica, Guadalajara C.P. 44430, Mexico; sandra.ruiz@academicos.udg.mx; 7 Instituto de Investigación en Enfermedades Crónico Degenerativas, Centro Universitario de Ciencias de la Salud, Universidad de Guadalajara, Calle Sierra Mojada No. 950, Colonia Independencia, Guadalajara C.P. 44340, Mexico; eloisa.ramos@academicos.udg.mx; 8 Laboratorio de Ciencias Morfológico-Forenses y Medicina Molecular, Departamento de Morfología, Centro Universitario de Ciencias de la Salud, Universidad de Guadalajara, Calle Sierra Mojada No. 950, Colonia Independencia, Guadalajara C.P. 44340, Mexico; josed.lopezj@academicos.udg.mx; 9 Centro de Investigación Biomédica de Occidente, Instituto Mexicano del Seguro Social, Calle Sierra Mojada No. 800, Colonia Independencia, Guadalajara C.P. 44340, Mexico

**Keywords:** exosomes, *miR-34a*, fat percentage

## Abstract

Extracellular vesicles (EVs), particularly exosomes, are key mediators of intercellular communication, transporting biomolecules such as nucleic acids, lipids, and proteins that influence immune and metabolic pathways. In adipose tissue (AT), adipocyte-derived EVs (AdEVs) play a crucial role in maintaining metabolic homeostasis and have been implicated in obesity-related dysfunction. Among their bioactive cargo, microRNAs regulate post-transcriptional gene expression and participate in immunometabolic regulation. This study aimed to determine whether *miR-34a* expression in serum and circulating EVs varies according to body fat percentage, to explore its potential utility as a non-invasive biomarker of AT dysfunction. A total of 142 adults (mean age 36 ± 11 years) were classified by body fat percentage (≥25% in men, ≥35% in women). Exosomes were isolated (Invitrogen^®^) and characterized by cryo-TEM, and *miR-34a* expression was quantified by qRT-PCR. *miR-34a* expression correlated negatively with Total Cholesterol, Triglycerides, LDLc/HDLc, TG/HDLc, BMI, C3, CRP, fasting insulin, HOMA-IR, HOMA-B, Body adiposity, Chemerin, CCL2, AdipoQT, and AdipoQ-H, but positively with HDLc and QUICKI. Notably, LDLc, sdLDLc, sdLDLc/LDLc, TC/HDLc, and fasting glucose showed opposite correlation patterns between serum and exosomes. Overall, serum *miR-34a* levels were higher than in exosomes, suggesting its potential as a biomarker of metabolic dysfunction and insulin resistance.

## 1. Introduction

Exosomes are a specific class of extracellular vesicles (EVs), typically measuring between 50 and 150 nm in diameter. They are produced through a distinct biogenetic pathway and can be detected in a variety of biological fluids—such as plasma, serum, urine, seminal fluid, tears, saliva, breast milk, and aqueous humor—as well as in different cell types and cultured media [1,2,3]. Exosomes play a crucial role in intercellular communication by transporting diverse biological materials, including nucleic acids (DNA, mRNA, microRNA), lipids, proteins and virulence factors, between cells [4,5]. Through the selective transfer of these molecules, exosomes contribute to the crosstalk between metabolic and immune networks. Their cargo not only mirrors the metabolic reprogramming of the parent cell but also redefines signaling cascades in recipient cells, ultimately shaping their immunometabolic phenotype [6,7].

In its secretory function, the AT (adipose tissue) is currently recognized as an essential source of EVs, also known as adipocyte-derived extracellular vesicles (AdEVs), which function as a bridge between adipocytes and cells in the stromal fraction of the AT as well as with cells from other systems [8]. AdEVs are filled with biological material that, in AT, play a role in metabolic alterations such as obesity, type 2 diabetes, and related illnesses and maintain the body’s homeostasis [9].

Behind the biological material are the microRNA (miRNAs or miRs), non-coding RNA of 18–25 nucleotides, which regulate gene expression post-transcriptional. It has been reported that many miRs are secreted between AdEVs and within the AT [10,11,12,13,14,15,16]. In this regard, the family of *miR-34* is conserved in mammalian organisms and consists of three members: *miR-34a* and *miR-34b/c* are encoded in chromosomes 1 and 11, respectively [17]. *miR-34a* mostly expresses in adipocytes and macrophages, has been implicated in the regulation of immune and metabolic functions in AT, inhibiting M2 macrophage activity by downregulating KLF4 expression, and is positively associated with insulin resistance (IR) and indicators of metabolic inflammation [18,19].

The global prevalence of obesity is a growing concern, with projections estimating that up to 40% of adults will be classified as overweight or obese by 2025 [20]. The primary indicator for obesity is the body mass index (BMI; >30 kg/m^2^) [21]. However, BMI does not provide enough information about the status of AT (hyperplasia and hypertrophy [22]), which becomes dysfunctional in obesity. Elevated levels of proinflammatory markers and imbalances in adipokines exacerbate this dysfunction.

Based on recent evidence identifying miRs as promising biomarkers in serum and EVs [23,24], and considering that epigenetic mechanisms help explain how hereditary and environmentally acquired factors contribute to the global rise in obesity [25], the aim of this study was to evaluate whether its expression in serum and circulating exosomes differs between adults with normal and high body fat. Comparing these two miRs sources allows us to explore whether changes in circulating *miR-34a* reflect early immunometabolic alterations related to adipose tissue expansion. Furthermore, we assessed its associations with metabolic, inflammatory, lipid, and anthropometric markers, with particular emphasis on differences according to body fat percentage.

## 2. Results

Individuals aged 20 to 59 years, 84 women and 58 men, were included in the study. There was an increment in blood pressure, inflammatory parameters, insulin resistance status, and the body adiposity status in the high-fat group versus normal-fat percentage subjects, especially in the visceral area which is reflected in the abdominal measures and indices (Table 1).

Serum and exosome-derived *miR-34a* showed distinct correlation patterns across lipid, inflammatory, insulin-resistance, adiposity, and adipokine parameters. The observed correlations indicate a tendency that, in our initial assessment, warrants consideration. Firstly, serum *miR-34a* correlated positively with HDLc. We noted opposite trends between compartments for other lipids: serum *miR-34a* showed a positive correlation with sdLDLc, whereas exosomal *miR-34a* displayed a strong negative correlation. Both serum and exosomal *miR-34a* correlated negatively with triglycerides. Cardiovascular risk ratios showed correlations in both compartments. Serum *miR-34a* exhibited positive correlation with the TC/HDLc ratio and negative with the TG/HDLc ratio. In contrast, exosomal *miR-34a* correlation was negative with the LDLc/HDLc ratio. Secondly, inflammatory parameters also showed negative correlations: C3 with serum *miR-34a*, while CRP and AdipoQT with exosomal *miR-34a*. Regarding insulin resistance status, serum *miR-34a* exhibited negative correlation with HOMA-B, while QUICKI showed a positive correlation. Third, negative correlations were observed for serum *miR-34a* during body adiposity status evaluation, specifically concerning BMI and AVI. Finally, serum *miR-34a* correlations with adipokines were close to zero, whereas exosomal *miR-34a* displayed a negative correlation with AdipoQT (Table 2).

To evaluate the diagnostic potential of serum and exosomal *miR-34a* expression, we performed univariate and multivariate logistic regression analyses incorporating insulin resistance (HOMA-IR), cardiovascular risk (sdLDL-c), and body fat percentage as dependent variable. As shown in Table 3, Models 1 and 2 exhibited non-significant *p*-values (*p* = 0.11 and *p* = 0.70, respectively) and low explanatory power (R^2^ = 0.30 and 0.01), with AUC values of 0.6. Model 3 showed the highest explanatory capacity (R^2^ = 0.60) and an AUC of 0.9, although its *p*-value remained non-significant (*p* = 0.08). Model 4 displayed moderate fit (R^2^ = 0.20) with an AUC of 0.6. Overall, none of the models reached statistical significance.

**Figure 1:**
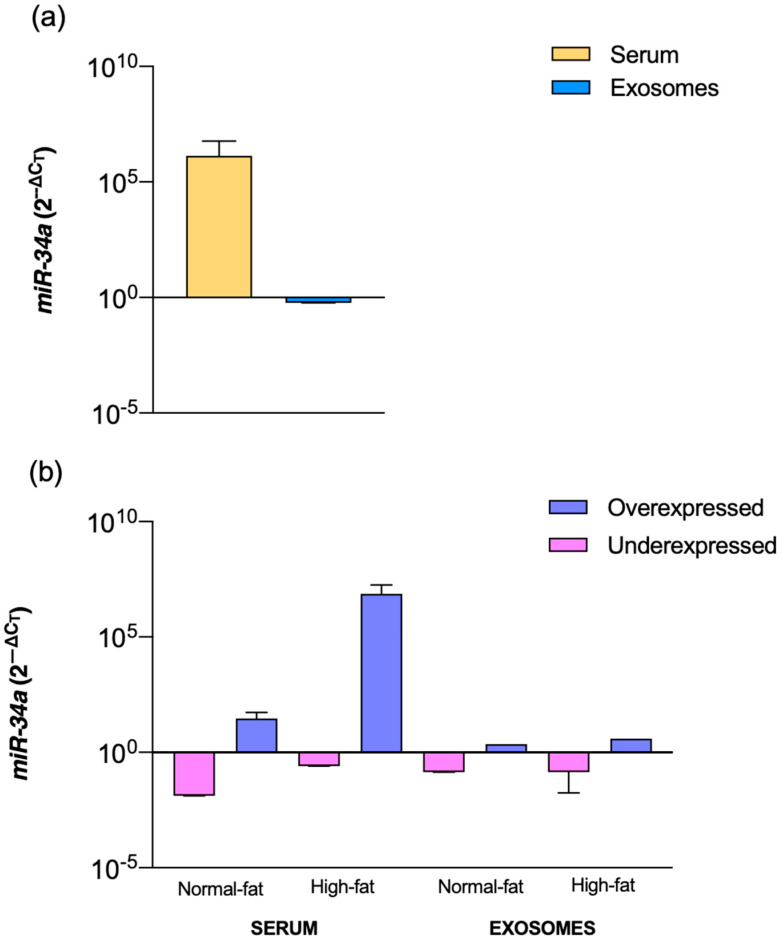
Serum and exosome *miR-34a* relative expression in subjects with normal and high-fat percentage (**a**) *miR-34a* expression in serum versus exosomes, (**b**) under and overexpression of *miR-34a* in serum and exosomes in normal and high-fat percentage subjects. Mann–Whitney U test, Kruskal-Wallis test, and Dun’s multiple comparison test. No statistically significant differences were found.

To evaluate whether *miR-34a* expression differed between individuals with normal and high fat percentages, we first compared its relative expression in serum and circulating exosomes. As shown in Figure 1a, *miR-34a* levels were markedly higher in serum than in exosomes across the study population. When participants were classified by body fat percentage, a similar pattern was observed: serum samples consistently showed higher *miR-34a* expression, whereas exosomal levels remained lower (Figure 1b). In both normal-fat and high-fat groups, individuals exhibited a mixed distribution of overexpression and underexpression, indicating that both expression patterns were present within each category. However, the overall trend persisted, with serum displaying higher *miR-34a* expression than exosomes regardless of adiposity status.

## 3. Discussion

Deciphering the role of epigenetic regulation mediated by exosomal microRNAs in metabolic health and disease could significantly enhance our understanding of the molecular mechanisms underlying the dysregulation of inflammatory and metabolic pathways in obesity. This understanding could pave the way for developing novel therapeutic approaches and predictive biomarkers to address obesity-related disorders.

Addressing the inflammation-associated *miR-34a*, it has been reported that correlated with proinflammatory markers, such as CXCL9, TNF, and IL10, and is a senescence-associated microRNA, characterized by its increased expression in serum and various tissues [26,27,28]. This investigation was performed in serum or tissue, while we, for the first time, compared *miR-34a* in serum and circulating exosomes in the fat percentage context, considering that a portion of circulating exosomes derived from adipose tissue [29].

Our findings revealed comparable correlation patterns between serum and exosomal *miR-34a* and the inflammatory and adipokine markers evaluated. The negative correlations observed for CRP and AdipoQ-H are consistent with previous reports in individuals with obesity and metabolic dysfunction, as described by Lischka et al. [30], suggesting that their association with *miR-34a* may reflect inflammatory status rather than fat mass itself. The negative correlation observed here may relate to the locally involvement of *miR-34a* in signaling pathways upstream of adipokine regulation, particularly in the activation of inflammatory pathways mediated by NF-κB [31,32]. Specifically, chemerin acts as a proinflammatory adipokine that activates NF-κB signaling through its receptor CMKLR1, promoting the secretion of cytokines such as IL-6 and TNF-α within adipose tissue [33]. *miR-34a* modulates this pathway by targeting SIRT1, a deacetylase that normally suppresses NF-κB activity. Downregulation of SIRT1 by *miR-34a* leads to enhanced acetylation and activation of the NF-κB subunit p65, thereby amplifying chemerin-induced inflammatory signaling [32]. Furthermore, *miR-34a* may also directly regulate NF-κB pathway components such as IκBα, contributing to a sustained proinflammatory state in obesity [34,35]. This crosstalk positions *miR-34a* as a key epigenetic amplifier linking adipokine signaling to chronic inflammation in expanded adipose tissue. Cheleschi et al. [35] demonstrated that *miR-34a* participates in NF-κB modulation within visfatin signaling, providing a plausible mechanistic link connecting *miR-34a*, inflammation, and adipokine dynamics. Taken together, these findings support a coherent immunometabolic framework in which *miR-34a* interacts with inflammatory and adipokine-related pathways.

*miR-34a* plays a crucial role in lipid and glucose metabolism by inhibiting SIRT1 and downregulating HNF4, two key regulators of these metabolic pathways [36,37]. Consistent with existing literature, we demonstrated for the first time a correlation between serum and exosomal *miR-34a* expression in individuals with normal and high fat percentages. Notably, exosome *miR-34a* exhibited a significant correlation with lipids, strongly with sdLDLc. Contrary with our findings, Li et al. [38], reported positive correlations of *miR-34a* with triglycerides and total cholesterol and a negative correlation with HDLc in patients with an existence heart disease. Similarly to our results, Shen et al. [39] observed that *miR-34a* positively correlates with LDLc and negatively with triglycerides in individuals with type 2 diabetes mellitus (T2DM). For exosomal *miR-34a*, our results have an opposites trend with those of Alshaymaa et al. [40] who reported positive correlations with the lipid profile in children with T1DM, while in our study showed a negative correlation with most of the lipid markers in metabolic healthy individuals. These findings provide further evidence of *miR-34a*’s involvement in lipid regulation and its potential as a biomarker in metabolic disorders.

In this context, several cardiovascular disease (CVD) risk ratios have been identified as markers for both metabolic syndrome and CVD. As reported by Kosmas et al., the TG/HDLc ratio has been suggested as a valuable predictor for various aspects of CVD [41]. Similarly, an elevated LDLc/HDLc ratio has been linked to the presence of carotid plaques, indicating a higher risk of atherosclerosis [42]. In our study, we observed a positive correlation between the sdLDLc/LDLc ratio and the expression of serum *miR-34a*, further supporting the role of this miRNA in lipid metabolism. This finding aligns with previous reports suggesting that *miR-34a* plays a key role in the regulation of lipid homeostasis, potentially influencing pathways related to cholesterol transport and atherogenesis. The association between sdLDLc, a well-known atherogenic lipid fraction, and *miR-34a* expression may provide additional insights into the molecular mechanisms underlying dyslipidemia and its contribution to metabolic and cardiovascular risk [36,37].

As previously discussed, *miR-34a* plays a pivotal role in glucose metabolism, influencing not only within the AT, but also on visceral fat accumulation and hepatic processes by targeting genes such as SIRT1, fibroblast growth factor 21 (FGF21), nicotinamide phosphoribosyl transferase (NAMPT), and ENO3, effectively implicated in high-fat diet-induced insulin resistance (IR), where overexpression of hepatic *miR-34a* lowered insulin signaling and altered glucose metabolism [43]. The tissue-specific targets of *miR-34a* further elucidate its systemic metabolic impact. In adipose tissue, *miR-34a* represses SIRT1 and KLF4, modulating macrophage polarization and insulin sensitivity [19]. In the liver, it directly targets HNF4α, ENO3, and NAMPT, disrupting gluconeogenesis and lipid homeostasis [37,43]. In skeletal muscle, *miR-34a* regulates FGF21 and VAMP2, affecting glucose uptake and insulin secretion [44,45]. These coordinated actions across metabolic tissues underscore *miR-34a*’s role as a pleiotropic regulator integrating inflammatory and metabolic signals in obesity. Our findings reveal a negative correlation between *miR-34a* and all IR parameters, contrary with the report by Pan et al. [19], who demonstrated a correlation between *miR-34a* and HOMA-IR in AT, leading to glucose intolerance and IR. Additionally, HOMA-B was positively correlated with serum *miR-34a* isolated from PBMCs from individuals with T2DM, as reported by Shen et al. [39], these findings contrast with our observations in both serum and exosomal *miR-34a*. Notably, the suppression of vesicle-associated membrane protein 2 expression, a crucial component of β-cell exocytosis, has been connected to the exosomal *miR-34a* impact [45]. Studies examining the expression of *miR-34a* in the visceral adipose tissue (VAT) and subcutaneous adipose tissue (SAT) of people with IR and non-diabetic controls corroborate our findings, emphasizing the significance of adipose tissue depots in IR [46].

The expression of *miRs* has been extensively studied across various diseases, with promising findings reported in serum, tissue, and cell-derived exosomes [47,48]. In this study, we focused on serum and circulating exosomal *miR-34a*. *miR-34a* expression in AT is significantly lower in normal weight persons but increases under obese situations due to metabolic stress in adipocytes [49]. Our data showed a similar trend for serum *miR-34a*, although no significant differences were found. This shows that *miR-34a*’s effects are essentially local in the growth and development of dysfunctional AT. This idea is consistent with the findings of Pan et al., who reported variations in exosomal *miR-34a* expression in VAT and SAT between lean and overweight/obese people [19].

Current literature primarily emphasizes the role of *miR-34a* in obesity-related disorders, with limited investigation in individuals without clinical disease but exhibiting different body fat percentages. Based on our findings, we propose that *miR-34a* may serve as a biomarker of adipose tissue dysfunction and a potential indicator of early metabolic alterations associated with excess adiposity. Moreover, considering its systemic immunometabolic involvement, future approaches may benefit from evaluating *miR-34a* in combination with other microRNAs, as suggested by previous studies using composite miRNA signatures to improve biomarker performance.

In the context of predictive performance, none of the logistic regression models reached statistical significance, despite Model 3 showing a higher explanatory capacity (R^2^ = 0.60) and an AUC of 0.9. These findings indicate that *miR-34a* expression alone is insufficient to reliably discriminate individuals with normal versus high fat percentage. This contrasts with studies performed in populations with established metabolic or other diseases, where *miR-34a* demonstrates stronger predictive utility [50,51,52]. Our results suggest that in metabolically healthy individuals, *miR-34a* may reflect early immunometabolic changes but does not, by itself, provide adequate sensitivity or specificity for diagnostic use. Future models incorporating combined miRNA signatures, together with metabolic and inflammatory markers, may enhance predictive performance.

Additionally, although oxidative stress markers were not measured in our study, the available literature indicates that *miR-34a* participates in oxidative stress-related pathways [37,53,54], adding another layer through which this miRNA may influence metabolic deterioration. Future research should explore this connection in greater depth, as targeting *miR-34a*–related oxidative and immunometabolic pathways may offer promising therapeutic opportunities for addressing early stages of obesity-associated dysfunction.

## 4. Materials and Methods

### 4.1. Samples

One hundred forty-two individuals aged 20 to 59 (58 men and 84 women) were classified by fat percentage; a high fat percentage was considered more than 25% in men and 35% in women, and we included 74 individuals with normal and 68 with high fat percentages. In fasting conditions, 10 mL of blood was obtained in EDTA tubes for exosome isolation, and without anticoagulant tubes for all the tests. The samples were allowed at room temperature for 30 min and were centrifuged at 3000× *g* for 15 min at room temperature to separate plasma and serum. Blood collection was approved by the Comisión de Investigación y Ética del Antiguo Hospital Civil de Guadalajara “Fray Antonio Alcalde”, Guadalajara, México. O.P.D. HCG/CEI-0835/22, NO. 130/22, and participants provided written informed consent.

### 4.2. Chemical and Anthropometric Indices

The following tests were performed according to the manufacturer’s recommendations: glucose (mg/dL), basal serum insulin (μΙU/mL), total adiponectin (AdipoQT), adiponectin of high molecular weight (AdipoQ-H), chemerin, and CCL2 by immunoassay type ELISA. Lipid profile (mg/dL): total cholesterol, triglycerides, HDLc, LDLc and sdLDLc were performed by the immunoturbidimetric method, and VLDLc was calculated by the Friedewald formula. Anthropometric indices (BMI [55], BAI [56], AVI [57], CI [58], VAI [59], and WC [59]) were calculated as mentioned before.

### 4.3. Exosome Isolation and Characterization

The Total Exosome Isolation Kit (Invitrogen^®^, Cat. No. 4404450, Vilnius, Lithuania) was used to isolate exosomes from plasma as recommended. Exosome isolation and characterization in the present study represent a methodological continuation of the standardization previously reported by Noboa et al. [60]. In that work, vesicles were characterized by Western blot detection of the tetraspanins CD81 and CD9 and by transmission electron microscopy (TEM) using a FEI Tecnai Spirit BioTwin microscope (FEI Technology; FEI TIA software v4.15, Hillsboro, OR, USA). Throughout the manuscript, the term ‘exosomes’ is used to refer to small extracellular vesicles enriched by the isolation method employed. This terminology is used in an operational sense and does not imply definitive attribution of endosomal biogenesis, in accordance with MISEV 2023 recommendations [3].

### 4.4. miRs Extraction from Isolated Exosomes and Serum

Isolated exosomes were lysed by Radioimmunoprecipitation assay buffer (RIPA), and briefly Trizol reagent was used to extract total miRNA. The total miRs concentration was assessed using a Qubit assay kit Invitrogen™. cDNA was synthesized with a cDNA Synthesis Kit (TaqMan™ Advanced miRNA cDNA Synthesis Kit, Carlsbad, CA, USA). *miRNA* relative expression was performed using SYBRGreen Master Mix (Applied Biosystem, Warrington, UK) in real-time qRT-PCR (StepOne, Foster City, CA, USA). Normalized to the mean of CT of *miR-24-3p* that served as internal control [61]. Obtained −2^ΔC^_T_ values give the logarithmic relative expression. The *miR-24-3p* and *miR-34-5p* primers used in PCR were extracted with T4Oligo (Irapuato, Gto, Mexico) (primer assay *miR-24-3p* CTGCTGAACTGAGCCA, *miR-34-5p* AGCTAAGACACTGCCA, both with 10X MiniScript (Quiagen, Hilden, Germany)).

### 4.5. Statistical Analysis

The differences were analyzed using the Mann–Whitney U test for the serum and exosome microRNA concentrations. The correlation was determined by the Spearman rho test. Data are presented as mean ± standard deviations, with statistical significance set at *p* < 0.05. GraphPad Prism version 8.4.0 for macOS was used for data analysis and graphing.

## 5. Conclusions

This study aimed to evaluate the presence and differential expression of *miR-34a* in serum and plasma exosomes among adults characterized by normal or increased body fat percentage. We observed the presence of *miR-34a* in both compartments, and the expression levels exhibited alternately divergent and specific relative expression and correlation patterns with markers of metabolism, inflammation, insulin resistance status, and body adiposity.

In this context, we highlight the importance of three aspects. First, we observed an evident negative correlation between serum sdLDLc concentration and exosomal *miR-34a* overexpression in individuals with increased body fat percentage, and second, we noted a tendency toward negative correlation with parameters such as triglycerides, the LDLc/HDLc ratio, and AdipoQT. Third, in contrast, a tendency toward positive correlation was observed between the relative expression levels of serum *miR-34a* and the concentration of HDLc and the TC/HDLc, BMI, and AVI ratios, as well as a negative correlation with C3.

The divergence in these observations suggests that the relative expression of *miR-34a* in both compartments—serum and exosomes—may reflect early alterations in lipid metabolism. Furthermore, it may indicate a subclinical inflammatory process developing toward chronicity, preferentially in individuals with an increased body fat percentage.

We recommend future studies to define the relevance of the relative expression of *miR-34a* and other miRs as potential targets.

## Figures and Tables

**Table 1:** Clinical and anthropometric characteristics of study subjects.

**Groups**	**Normal-Fat Percentage**	**High-Fat Percentage**	***p*** **Value**
n	74	68	
Sex (Men:Women)	33:41	25:43	
Age (years)	40 ± 9	33 ± 11	
**Blood Pressure (mmHg)**			
Systolic	109 ± 7	117 ± 13	**0.0015**
Diastolic	67 ± 11	75 ± 11	**0.0003**
**Lipid Profile (mg/dL)**			
HDLc	35.6 ± 13.9	33.3 ± 6.8	NS
LDLc	93.4 ± 47.9	108.2 ± 56.1	NS
sdLDLc	21.8 ± 6.1	29.3 ± 18.9	NS
VLDLc	22.4 ± 13.8	40.6 ± 38.1	**<0.0001**
TC	176 ± 46	196 ± 45	**0.0299**
Triglycerides	107 ± 71	207 ± 189	**<0.0001**
**Cardiovascular risk ratios**			
sdLDLc/LDLc	0.4 ± 0.4	0.5 ± 0.7	NS
TC/HDLc	5.2 ± 1.5	6.1 ± 2.9	**0.0003**
LDLc/HDLc	2.8 ± 1.4	3.4 ± 2.3	NS
TG/HDLc	3.1 ± 1.9	6.8 ± 8.4	**<0.0001**
**Inflammatory Parameters**			
C3 (mg/dL)	75.9 ± 12.2	94.6 ± 17.4	**<0.0001**
CRP (mg/L)	5.7 ± 3.4	10.6 ± 7.0	**<0.0001**
**Insulin Resistance Status**			
Fasting glucose (mg/dL)	76.7 ± 16.5	87.7 ± 40.2	**0.0331**
Fasting insulin (μUI/mL)	12.4 ± 9.2	21.7 ± 11.2	**<0.0001**
HOMA-IR	2.5 ± 2.6	4.7 ± 4.1	**<0.0001**
HOMA-B	549 ± 878	6812 ± 1106	NS
QUICKI	0.35 ± 0.05	0.31 ± 0.02	**<0.0001**
**Body Adiposity Status Evaluation**			
BMI (kg/m^2^)	23.7 ± 4.3	33.8 ± 5.5	**<0.0001**
BAI	27. 6 ± 4.2	34.8 ± 9.5	**<0.0001**
AVI (cm^2^)	14.0 ± 5.8	23.0 ± 8.6	**<0.0001**
CI	15.9 ± 44.7	5.3 ± 32.4	**<0.0001**
VAI	1.3 ± 3.2	0.2 ± 0.1	**<0.0001**
WC (cm)	79.1 ± 18.5	103.7 ± 21.6	**<0.0001**
SA	126 ± 593	−401 ± 1524	NS
VA	394 ± 581	1294 ± 1556	**<0.0001**
**Adipokines (ng/mL)**			
AdipoQT	7055 ± 3683	6091 ± 3010	NS
AdipoQ-H	1672 ± 1487	1283 ± 1443	NS
Chemerin	112 ± 73	106 ± 52	NS
CCL2	0.3 ± 0.2	0.3 ± 0.2	NS

Mann–Whitney *U* test. *p* < 0.05 of significance. Abbreviations: NS, non-significative; HDLc, high-density, LDLc, low-density, sdLDLc, small low-density and VLDLc, very low-density lipoprotein cholesterol; TC, total cholesterol; CRP, C-reactive protein; HOMA-IR, homeostasis model assessment-estimated insulin resistance; HOMA-B, homeostasis model assessment of β-cell function; QUICKI, quantitative insulin-sensitivity check index; BMI, body mass index; BAI, body adiposity index; AVI, abdominal volume index; CI, conicity index; VAI, visceral adiposity index; WC, waist circumference; SA, subcutaneous adipose area; VA, visceral adipose area; AdipoQT, total adiponectin; AdipoQ-H, adiponectin of high molecular weight, CCL2, C-C motif chemokine ligand 2.

**Table 2:** Correlations of serum and exosome *miR-34a* relative expression.

Parameters	Serum *miR-34a* (rho)	Exosome *miR-34a* (rho)
**Lipid Profile** **(mg/dL)**		
HDLc	0.53	0.19
LDLc	0.17	−0.26
sdLDLc	0.35	−0.62 *
TC	−0.11	−0.27
Triglycerides	−0.42	−0.82
**Cardiovascular risk ratios**		
sdLDLc/LDLc	0.13	−0.06
TC/HDLc	0.42	−0.08
LDLc/HDLc	−0.07	−0.49
TG/HDLc	−0.60	−0.01
**Inflammatory Parameters**		
C3 (mg/dL)	−0.45	−0.05
CRP (mg/L)	−0.27	−0.39
**Insulin Resistance Status**		
Fasting glucose (mg/dL)	0.03	−0.24
Fasting insulin (μUI/mL)	−0.35	−0.25
HOMA-IR	−0.33	−0.27
HOMA-B	−0.39	−0.02
QUICKI	0.36	0.29
**Body Adiposity Status Evaluation**		
BMI (kg/m^2^)	−0.46	−0.01
BAI	−0.34	0.11
AVI	−0.55	−0.04
SA	−0.17	−0.13
VA	−0.13	0.04
**Adipokines** **(ng/mL)**		
AdipoQT	−0.05	−0.40
AdipoQ-H	−0.06	−0.04
CHEM	−0.06	−0.13
CCL2	−0.07	−0.35

Spearman rank correlation coefficients (rho). * *p* < 0.05 of significance. Abbreviations: HDLc, high-density, LDLc, low-density and sdLDLc small low-density lipoprotein cholesterol; TC, total cholesterol; CRP, C-reactive protein; HOMA-IR, homeostasis model assessment-estimated insulin resistance; HOMA-B, homeostasis model assessment of β-cell function; QUICKI, quantitative insulin-sensitivity check index; BMI, body mass index; BAI, body adiposity index; AVI, abdominal volume index; SA, subcutaneous adipose area; VA, visceral adipose area; AdipoQT, total adiponectin; AdipoQ-H, adiponectin of high molecular weight, CCL2, C-C motif chemokine ligand 2.

**Table 3:** Logistic regression for *miR-34a* relative expression.

Model	*p* Value	Nagelkerke R^2^	AUC
M_1_	0.11	0.30	0.6
M_2_	0.70	0.01	0.6
M_3_	0.08	0.60	0.9
M_4_	0.20	0.20	0.6

*p* < 0.05 of significance. M_1_ includes: ΔCt serum *miR-34* expression. M_2_ includes: ΔCt exosome *miR-34* expression. M_3_ includes: ΔCt serum *miR-34* expression, sdLDLc, HOMA-IR. M_4_ includes: ΔCt exosome *miR-34* expression, sdLDLc, HOMA-IR.

## Data Availability

The raw data supporting the conclusions of this article will be made available by the authors on request.
